# Role stress, social support and occupational burnout among physicians in China: a path analysis approach

**DOI:** 10.1093/inthealth/ihz054

**Published:** 2019-07-25

**Authors:** Hui Ma, HuiFen Qiao, HaiTao Qu, Hui Wang, Yun Huang, Hao Cheng, ChangJun Teng, KaiLi Diao, XiangRong Zhang, Ning Zhang

**Affiliations:** Department of Medical Psychology, The Affiliated Brain Hospital of Nanjing Medical University, Nanjing, China; Department of Medical Psychology, The Affiliated Brain Hospital of Nanjing Medical University, Nanjing, China; Educational and Counseling Department of Mental Health, Nanjing University of Posts and Telecommunications, Nanjing, China; Department of Neurology, Lianyungang Hospital of Traditional Chinese Medicine, Lianyungang, China; Medical Insurance Audit Department, Hangzhou Medical Insurance Management and Service Bureau, Hangzhou, China; Educational and Counseling Department of Mental Health, Nanjing University of Posts and Telecommunications, Nanjing, China; Department of Medical Psychology, The Affiliated Brain Hospital of Nanjing Medical University, Nanjing, China; Psychology Department, Nanjing Normal University, Nanjing, China; Department of Medical Psychology, The Affiliated Brain Hospital of Nanjing Medical University, Nanjing, China; Department of Medical Psychology, The Affiliated Brain Hospital of Nanjing Medical University, Nanjing, China

**Keywords:** Chinese physicians, occupational burnout, role stress, social support

## Abstract

**Background:**

Occupational burnout in physicians is prevalent and can have many negative effects. The purposes of this study were to explore the prevalence of occupational burnout and to analyze the effects of social support and role stress on occupational burnout among Chinese physicians.

**Methods:**

Using multistage-stratified cluster random sampling, physicians were selected to participate in the study and completed three questionnaires: the Chinese Maslach Burnout Inventory; the Cross-Cultural Role Conflict, Ambiguity and Overload Scale; and the Social Support Rating Scale. A path analysis was run to test the effects of role stress and social support on occupational burnout.

**Results:**

Of 2530 physicians, 864 (34.2%) were experiencing moderate occupational burnout and 140 (5.5%) were experiencing severe occupational burnout. The path analysis results indicated that role conflict had direct positive effects on emotional exhaustion (EE) and depersonalization (DP), and role ambiguity had direct positive effects on DP and decreased personal accomplishment (DPA). Coworker support had direct negative effects on EE and positive effects on DP, family support had direct negative effects on DP and DPA. Coworker support mediated the effects of role ambiguity on EE and DP, and family support mediated the effects of role ambiguity on DP and DPA.

**Conclusions:**

These findings suggest that occupational burnout is common in Chinese physicians, and that role stress and social support play important roles in occupational burnout. Interventions that aim to reduce role stress and increase social support can be effective approaches to prevent occupational burnout among physicians.

## Introduction

In 1974, the American psychoanalyst Herbert J. Freudenberger first used the term ‘occupational burnout’ to refer to the state of exhaustion and fatigue resulting from persistent emotional input by individuals working for others.^1^ In 2001, Maslach, Schaufeli and Leiter used three aspects to assess occupational burnout: emotional exhaustion (EE), depersonalization (DP) and decreased personal accomplishment (DPA).^2^

According to data from the International Congress of Psychology, ‘physician’ is a high-risk occupation in which occupational burnout is prevalent. Research has shown that 25–60% of physicians are in a state of occupational burnout, and this proportion reached 75% in some studies.^3,4^ Recently, with the reform of medical systems, changes in the medical environment and growing work pressure, occupational burnout has shown an increasing trend among physicians.^5,6^ Occupational burnout may not only induce psychosomatic symptoms, such as insomnia, anxiety, depression and gastrointestinal dysfunction, but also leads to loss of enthusiasm for work, decreased job satisfaction, quitting and early retirement, depression, and even death among physicians.^3,7,8^ Furthermore, occupational burnout in physicians was reported in association with higher incidences of inappropriate treatment of patients and more malpractice, which decrease patients’ satisfaction and compliance with treatment.^3,9^

In China, clinicians are overloaded and under great work pressure. Demands on doctors have increased with health system reform involving gradual replacement of the traditional physician-centric model with a patient-centered model. In 2011, 1.49 doctors were available for every 1000 people in China (China Health Statistics Yearbook, 2012), which is considerably less than in western countries.^10^ Inefficient work environments, excessive workloads, loss of support from colleagues and superiors, and work-family conflicts have been associated with occupational burnout in Chinese physicians.^11,12^ Reported rates of moderate to severe occupational burnout among hospital physicians in China are 66.5–74.0%.^12,13^ However, these rates may not be representative of the overall level and prevalence of occupational burnout among Chinese physicians because of the examination of small samples, from single cities or types of hospital, in previous studies.

Exploration of the specific mechanisms underlying occupational burnout is very important to gain a full understanding and take effective measures to prevent it. The Job Demand-Resources (JD-R) model was developed to aid understanding of the mechanisms through which job demands and resources (as working conditions found in all organizational contexts) are related to occupational burnout.^14^ Role stress and social support are two important influencing factors in the JD-R model. Role stress, including role conflict and role ambiguity, has been identified as an important work-related stressor associated strongly with occupational burnout.^15^ Role stress can also enhance individuals’ internal tension and frustration, and lead to the occurrence of occupational burnout.^16^ Social support is a job resource that can not only directly reduce the level of occupational burnout, but also serves as a buffer, effectively alleviating the impact of role stress. Social support from colleagues and superiors was found to be negatively related to occupational burnout, and to reduce the burnout level.^17^ Some studies have shown that role stress and social support are related independently to occupational burnout in China,^18^ but the combined effect of role stress and social support on occupational burnout have not been examined.

In this study, we aimed to quantify the degree of occupational burnout and investigate the correlations of role stress and social support from different sources with occupational burnout, and the potential moderating effects of social support on the relationships between role stress and symptoms of occupational burnout among physicians in China.

## Materials and methods

### Subjects and procedure

The sample size was calculated according to the formula:^19^


$$n=\frac{Z_{1-\alpha /2}^{2}\overset{L}{\underset{h=1}{\sum }}\left[N_{h}^{2}P_{h}(1-P_{h})/W_{h}\right]}{N^{2}d^{2}},$$

where n is the sample size, *N*_*h*_ is the number of physicians on each level and N is the number of national physicians, *W*_*h*_ is the proportion of *N*_*h*_ in N and *P*_*h*_ is the incidence of occupational burnout at each level; d is the desired level of precision, which we set at 0.02; *α* was set at 0.05, namely, $Z_{1-\alpha /2}=1.96$. According to the data from an initial investigation undertaken in Jiangsu Province,^13^ we assumed *P*_*h*_ is 0.7, according to the formula, $n=\frac{1.96^{2}\times 0.7\times (1-0.7)}{0.02^{2}}=2017$.

That is to say, the sample size in the actual survey was calculated to be no less than 2017 individuals. Three regions (eastern, central and western), defined by the China Health Statistics Yearbook,^20^ were used as the stratification standard in the first stage to randomly select 11 provinces (Jiangsu, Anhui, Liaoning, Hunan, Shanxi, Henan, Heilongjiang, Sichuan, Guizhou, Yunnan and Xinjiang). In the second stage, the grading of medical institutions was used as the stratification standard to randomly select 15 hospitals in each province. In the hospital grading system of China’s Ministry of Health, all medical institutions are classified as primary, secondary or tertiary hospitals based on their functions and medical resources. In the last stage, the sample group was selected from those hospitals by simple random sampling. In eastern China, we took the hospital as the unit for implementation of a direct sample survey, with researchers distributing, interviewing and collecting questionnaires on site. In the central and western regions, we mailed questionnaires to personnel designated as being responsible for questionnaire distribution, collection and return. All respondents were practicing doctors certified by the Ministry of Health, People’s Republic of China, who had worked at their current hospitals for no less than 1 y. In China, all physicians must complete a 6-mo to 1-y probation period, after which they may become full-time physicians.

The Ethics Committee of the Affiliated Brain Hospital of Nanjing Medical University approved this study (no. 2008-KY06). All individuals provided written informed consent before participation after receiving a full explanation concerning the study. Clinically trained personnel performed individual and collective measurement using identical instructions and also collected questionnaires. For the protection of privacy, the questionnaires were anonymous.

### Demographics

Demographic data collected from each respondent included gender, age, marital status and years of practice. Age was classified as being aged <25, 25–34, 35–44, 45–54 or ≥55 y. Marital status was categorized as single, partnered (married or a partner) or divorced/widowed. In this study, only 43 participants (1.7%) belonged to the divorced/widowed group, so when we compared occupational burnout between groups, this group was combined with the single group. Years of practice was classified as <5, 5–9, 10–19 or ≥20 y.

### Chinese Maslach Burnout Inventory

The Chinese Maslach Burnout Inventory (CMBI),^21^ adapted from the Maslach Burnout Inventory and shown to be suitable for respondents with Chinese cultural backgrounds, was used to assess physicians’ occupational burnout. The scale consists of 15 items in three dimensions (EE, DP and DPA; five items for each). Each item is ranked on a seven-point Likert scale ranging from 1 (never) to 7 (every day). Higher scores reflect greater occupational burnout. Occupational burnout was divided into four levels (none, slight, moderate and severe) according to the number of scores meeting or exceeding threshold values (EE≥25, DP≥11 and DPA≥16, zero to three, respectively). No score met the threshold values means none occupational burnout, one number of three scores met or exceeded threshold values means slight occupational burnout, two numbers of three scores met or exceeded threshold values means moderate occupational burnout, and all three scores met or exceeded threshold values means severe occupational burnout. The reliability and validity of the CMBI have been proven.^21^

### Role stress

The Cross-Cultural Role Conflict, Ambiguity and Overload scale^22^ was also used. The complete scale consists of 13 items; 5 items were used to assess individual role ambiguity, 3 items were used to assess individual role conflict, and 5 items were used to assess individual role overload. Each item is ranked on a five-point Likert scale ranging from 1 (very opposed) to 5 (very much agree). The scale of the Chinese version showed good reliability and validity.^18^ In this study, role ambiguity and role conflict were used, the Cronbach’s *α* coefficients for the two factors were 0.676 and 0.822, respectively.

### Social support

Social support was assessed using the Social Support Rating Scale^23^, which characterizes staff members’ perceived support from colleagues, supervisors, spouses, family members and friends. This scale is used widely and is one of the most stable scales for the assessment of social support in the work environment. The scale consists of three subscales: supervisor support, colleague support and family support. Each subscale contains four items, ranked on a five-point Likert scale ranging from 0 (never) to 4 (very much). The scale of the Chinese version showed good reliability and validity.^18^ In this study, the Cronbach’s *α* coefficients for supervisor support, colleague support and family support were 0.805, 0.736 and 0.753, respectively, which exhibited acceptable internal consistency.

### Data analysis

Data were analyzed using SPSS version 17.0 (SPSS Inc., Chicago, IL, USA), with a two-tailed significance level of at least p<0.05. Descriptive statistics were used to estimate the mean (X±SD) overall and dimensional incidences (%) of occupational burnout in Chinese physicians. Comparison between two groups of normally distributed data was performed using the two-sample t-test and analysis of variance, and comparison between two or more groups of non-normally distributed data was carried out using the Mann–Whitney U-test. Correlations between CMBI scores, role stress and social support scores were examined by Spearman bivariate correlation.

We estimated the weighted prevalence of occupational burnout on the basis of sampling weights and poststratifcation weights. The sampling weight was estimated according to the proportion of physicians in three regions. Poststratification weight was estimated on the basis of gender (male or female), age (<25, 25–34, 35–44, 45–54 or ≥55 y) and professional title (primary, intermediate or senior) using data from the China Health Statistics Yearbook (2009). The final weight for each participant was the product of sampling and poststratification weights.

A path analysis was performed using the maximum likelihood method with Amos 21.0 software (IBM Corporation Software Group, Somers, NY, USA) to test the effects of role stress and social support on burnout. Standardized path coefficients and the significance of direct, indirect and total effects were determined. The following indicators were used to examine the goodness of model fit: the ratio of χ^2^ to df (χ^2^/df), the goodness of fit index (GFI), the incremental fit index (IFI), the comparative fit index (CFI), the normed fit index (NFI) and the root mean square error of approximation (RMSEA). Satisfactory model fit is indicated by RMSEA values ≤0.10 and GFI, IFI, NFI and CFI values ≥0.90. χ^2^ tests as indicators of overall fit were omitted because of their sensitivity to sample size.

## Results

In total, 3000 questionnaires were delivered during December 2009 to February 2011, for which 2705 completed questionnaires were returned; 2530 of these returned questionnaires were valid (93.5% completion rate, 84.3% actual response rate). Of the 2530 responding physicians, 1234 (48.8%) were from hospitals in the eastern region, 643 (25.4%) were from the central and 653 (25.8%) were from the western region. The majority of respondents were male (n=1356 [53.6%]) and partnered (n=1962 [77.5%]). Respondent age ranged from 20 to 67 y. Nearly half (47.3%) of the physicians had worked for less than a decade (Table 1).

**Table 1.** Sociodemographic characteristics of participants

Variables	Demographic characteristics	Frequency (%)
Area	East	1234 (48.8)
	Central	643 (25.4)
	West	653 (25.8)
Gender	Female	1152 (45.5)
	Male	1356 (53.6)
	Not specified	22 (0.9)
Marital status	Single	502 (19.8)
	Partnered	1962 (77.5)
	Divorced/widowed	43 (1.7)
	Not specified	23 (1.0)
Age (y)	<25	113 (4.5)
	25–34	1233 (48.7)
	35–44	773 (30.6)
	45–54	318 (12.6)
	≥55	77 (3.0)
	Not specified	16 (0.6)
Years of practice	<5	638 (25.2)
	5–9	560 (22.1)
	10–19	832 (32.9)
	≥20	471 (18.6)
	Not specified	29 (1.2)

Mean EE, DP and DPA scores were 19.74±7.02, 11.41±6.47 and 15.46±5.70, respectively; weighting the dimensional incidences of occupational burnout, 22.1% of respondents reported severe EE, 37.3% reported severe DP and 40.9% reported severe DPA. In our samples, with weighting, the overall detection rate for occupational burnout was 61.3%; the prevalences of slight, moderate and severe burnout were 27.4% (n=869), 29.0% (n=864) and 5.0% (n=140), respectively. Univariate analysis of CMBI scores according to sociodemographic variables showed that males had significantly higher EE and significantly lower DPA scores than females (p<0.01 and p<0.05, respectively). Single physicians had significantly higher DP and DPA scores than those who were partnered (p<0.01). Compared with other age groups, EE scores were significantly lower among those aged 45–54 and ≥55 y; DP scores were significantly higher among those aged <25 y compared with other groups; DPA scores were significantly higher among those aged <25 and 25–34 y, and DPA scores were significantly lower among those aged ≥55 y than among those groups aged <55 y (Table 2).

**Table 2.** Univariate analysis of CMBI scores in relation to sociodemographic characteristics

Variables	CMBI scores (mean±SD)		
	EE	DP	DPA
Gender			
Male	20.18±7.13	11.56±6.51	15.16±5.79
Female	19.25±6.86	11.22±6.41	15.84±5.58
Z	−3.114^b^	−1.700	−3.194^a,b^
Marital status			
Single	20.05±7.25	12.28±6.69	16.36±5.65
Partnered	19.65±6.96	11.14±6.34	15.22±5.68
Z	−1.223	−4.132^b^	−4.128^b^
Age (y)			
<25	19.95±7.41	13.69±7.38	16.27±5.62
25–34	20.12±7.13	11.17±6.19	15.93±5.51
35–44	19.76±6.87	11.57±6.62	15.28±5.88
45–54	18.60±6.84	11.28±6.83	14.26±5.69
≥55	18.04±6.38	11.16±5.95	13.68±5.73
χ^*2*^	15.212^b^	12.104^a^	34.057^b^

CMBI: Chinese Maslach Burnout Inventory; DP: depersonalization; DPA: decreased personal accomplishment; EE: emotional exhaustion; Z: Statistics of nonparametric tests.

^a^p-value <0.05; ^b^p-value <0.01.

Role conflict and role ambiguity scores were significantly higher among physicians with than among those without occupational burnout (p<0.001). Scores for support from supervisors, coworkers and family members were significantly higher among physicians without than among those with occupational burnout (p<0.001; Table 3). Role ambiguity was correlated positively with EE, DP and DPA; role conflict was correlated positively with EE and DP, and negatively with DPA (p<0.01). EE was correlated negatively with coworker support (p<0.05) and was not correlated with supervisor or family support. All social support variables were correlated negatively with DP and DPA (p<0.01; Table 4).

**Table 3.** Mean role stress and social support scores

Variable	no occupational burnout	occupational burnout	Z
	N=657	N=1893	
Role conflict	8.46±2.83	9.38±2.74	−7.836^a^
Role ambiguity	9.30±3.73	11.98±4.19	−14.698^a^
Supervisor support	9.41±3.84	8.41±3.78	−6.166^a^
Coworker support	10.41±2.91	9.46±2.93	−7.801^a^
Family support	13.49±2.95	11.97±3.46	−11.492^a^

^a^p-value <0.001.

**Table 4.** Mean scores and correlations of role stress, social support and CMBI scores

Variables	Mean	SD	1	2	3	4	5	6	7	8
1. EE	19.74	7.02	-							
2. DP	11.41	6.47	0.227^b^	-						
3. DPA	15.46	5.70	−0.071^b^	0.341^b^	-					
4. Role conflict	9.14	2.79	0.336^b^	0.126^b^	−0.056^b^	-				
5. Role ambiguity	11.28	4.24	0.049^a^	0.352^b^	0.419^b^	−0.046^a^	-			
6. Supervisor support	8.67	3.82	0.005	−0.064^b^	−0.151^b^	−0.022	−0.202^b^	-		
7. Coworker support	9.70	2.96	−0.041^a^	−0.102^b^	−0.200^b^	0.004	−0.253^b^	0.598^b^	-	
8. Family support	12.36	3.40	−0.027	−0.307^b^	−0.325^b^	0.047^a^	−0.384^b^	0.220^b^	0.381^b^	-

DP: depersonalization; DPA: decreased personal accomplishment; EE: emotional exhaustion.

^a^p-value <0.05; ^b^ p-value <0.01.

According to the fitting result of the initial model by Amos, supervisor support was removed during the model revision process. The modified path model yielded the following values: χ^2^/df=4.986, RMSEA=0.040, NFI=0.983, CFI=0.986, GFI=0.994 and IFI=0.986. The RMSEA value was <0.10 and the fit indexes were >0.90, indicating an acceptable fit of the conceptual model (Table 5). These results indicate a relatively good fit of the path model of role stress, social support and occupational burnout among Chinese physicians.

**Table 5.** Fit index results for the path model

Model	χ^2^	χ^2^/df	NFI	CFI	GFI	IFI	RMSEA
optimal model	59.835	4.986	0.983	0.986	0.994	0.986	0.040

df: degrees of freedom; CFI: comparative fit index; GFI: goodness of fit index; IFI: incremental fit index; NFI: normed fit index; RMSEA: root mean square error of approximation.

**Figure 1.**
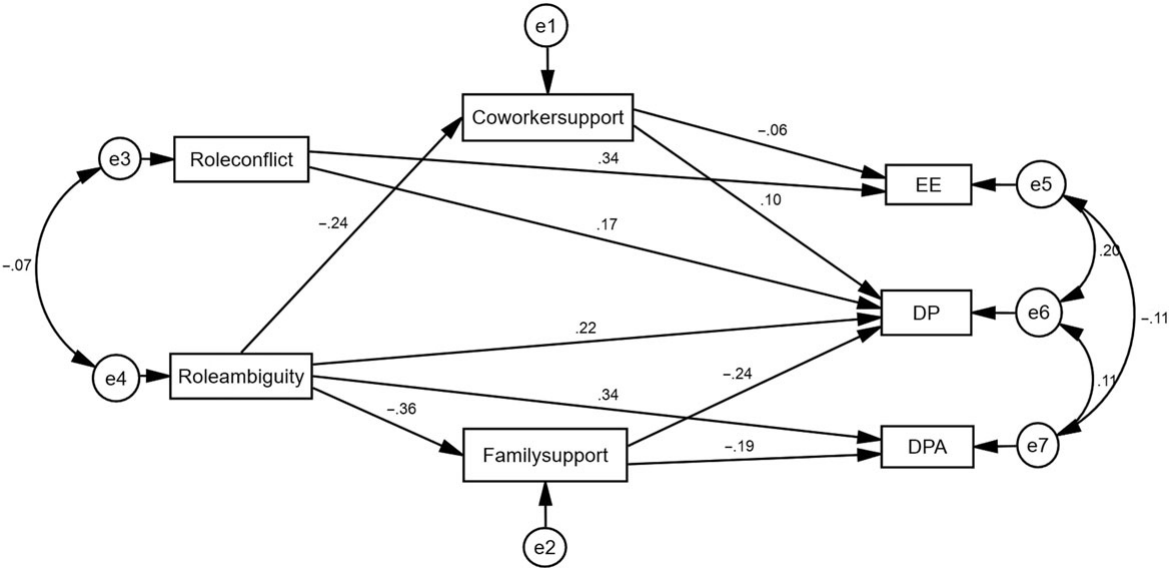
Path model of burnout among Chinese physicians. EE: emotional exhaustion; DP: depersonalization; DPA: decreased personal accomplishment.

The path model presented in Figure 1 shows that role conflict had direct positive effects on EE and DP, and that role ambiguity had direct positive effects on DP and DPA. Coworker support had direct negative effects on EE and positive effects on DP, while family support had direct negative effects on DP and DPA. Coworker support mediated the effects of role ambiguity on EE and DP, and family support mediated the effects of role ambiguity on DP and DPA. Of all the standardized total effects, the absolute value of role conflict on EE is the greatest (0.342). For the total effects on DP, the greatest factor is role ambiguity (0.285), followed by family support (−0.239), role conflict (0.167) and coworker support (0.105). Furthermore, the main factors on DPA are role ambiguity (0.408) and family support (−0.185).

## Discussion

In this study, 61.3% of Chinese physicians reported that they were experiencing occupational burnout, including 34.0% who reported moderate to severe occupational burnout. These results are similar to that of national data from other countries.^4,8^ Furthermore, the rate of DPA was much higher in this sample than in other countries.^24,25^ According to the occupational burnout development model proposed by Maslach, the first symptoms of occupational burnout reflect EE, followed by DP, which plays a protective role, and ultimately DPA. Thus, Chinese physicians are experiencing severe occupational burnout.

Recent research has shown that occupational burnout among physicians is related to factors such as work stress, workload and the organizational environment.^8^ According to the Chinese Medical Doctor Association’s fifth survey of physicians’ clinical status,^26^ 52.72% of physicians worked 40–60 h per wk on average and 32.69% of physicians worked >60 h per wk. More than half of physicians were not satisfied with their work environment or occupation and had low subjective well-being, and >60% of physicians expressed their unwillingness to support their children’s entry into medical professions. Moreover, their stress was derived mainly from excessive medical disputes, high patient expectations and the danger of physical abuse from patients and caretakers.^27^ Legal disputes such as malpractice suits are felt to be weighted against physicians, and this stressor also contributes to their DPA.

Similar to previous studies, we found that marital status, age and gender are related to occupational burnout.^12^ Single physicians experience more severe occupational burnout than married physicians do, especially with regard to DP and DPA. We also found that occupational burnout decreased gradually with age and that the prevalence of EE and DPA were substantially lower among physicians aged >45 y. Additionally, male Chinese physicians experienced more fatigue and burnout than female physicians and female physicians’ perceived personal accomplishments were less than those of males.

In this study, the level of role stress was higher among physicians with than among those without occupational burnout. Role conflict and role ambiguity are two factors that have been shown to be related to the different dimensions of occupational burnout, both in this and in previous studies.^15^ In China, physicians, especially those working in affiliated hospitals, are expected to take on many roles, such as clinical doctor, teacher and scientific researcher. These overlaps of multiple roles cause excessive pressure and role conflict. Moreover, physicians are unclear about goals for their roles and experience decreased self-evaluation, which influences their sense of self-value and interpersonal relationships. As a result, physicians suffer from indifferent interpersonal relationships and a low sense of achievement. These findings indicate that health sector managers should attempt to reduce role conflict and role ambiguity in physicians and prevent occupational burnout, such as reducing physicians’ work overload and improving their working conditions.

Social support is an important external resource that can prevent and reduce stress, alleviating occupational burnout. Previous studies have shown that social support from different sources is differentially related to burnout dimensions.^11,17,28^ Our study also showed that support from superiors, coworkers and family had significant negative relationships with DP and DPA, whereas only support from coworkers was related negatively to EE. Furthermore, analyzed by path model, different sources of social support have inconsistent impacts on the three dimensions of occupational burnout.

This study showed that support from coworkers mediated the relationships of role ambiguity to EE and DP, effectively easing physicians’ EE, but increasing the occurrence of DP. Coworkers and supervisors can reduce demands at work and also workloads, which is linked more closely to EE.^17^ However, organizational support does not enhance individuals’ positive images of themselves as physicians, which results in the dissemination of dissatisfaction when working directly with patients. Thus, tension between doctors and patients is increased, compromising the doctor–patient relationship and leading to physicians’ indifference, alienation, and even evasive attitudes when working with patients. This finding contributes to our understanding that the support of coworkers has a positive effect on preventing physicians’ occupational burnout. Strengthening relationships with coworkers may decrease occupational burnout and improve doctors’ quality of life.^11^

In this study, support from superiors was correlated negatively with DP and DPA but had no mediating effect on role stress in the path model. Thus, such support could not alleviate the occupational burnout caused by role stress, which is inconsistent with the majority of previous results.^17,29^ However, a study involving 494 Japanese physicians also showed that support from superiors had no effect on occupational burnout, whereas support from coworkers eased it.^28^ For Chinese physicians, the majority of such support comes from clinical department heads, but the receipt of such support cannot change the need to meet conflicting work requirements from departmental and hospital management. Therefore, physicians continue to face a variety of problems derived from role conflict and role ambiguity.

Family support plays an important role in social support but its impact on occupational burnout has not been well studied. A meta-analysis based on the conservation of resources model suggested that social support associated with work is related more closely to EE than support unassociated with work, which suggests that more research on relationships between personal resources and occupational burnout is needed.^17^ In this study, family support had significant negative relationships with DP and DPA, and mediated the relationships between these dimensions and role ambiguity, thereby preventing their occurrence. The family has always been a basic unit of Chinese society, and family support occupies an important position in the social network. With sufficient family support and harmonious family relations, individuals can be immersed in their work; if the family supplies little support, the addition of work pressure makes individuals prone to burnout. On the other hand, influenced by the current working environment and Chinese traditional culture, Chinese physicians tend to express negative emotions and negative effects related to work to their families. The receipt of comfort, encouragement and support from family members may help physicians to adjust their emotional state, reduce the negative impact on them and find positive coping strategies, thereby reducing the occurrence of occupational burnout.

This study has some limitations. First, data were collected by self-report questionnaire. We used an anonymous questionnaire in the hope that the physicians surveyed would reflect upon their own situations honestly. However, they could have exaggerated or minimized the indicators of occupational burnout, which would have biased the data. Second, the study was a cross-sectional design, which may lead to a causal effect with low power by path analysis. Longitudinal studies will be conducted to confirm our results in future work. Third, although a representative sample was selected, sample weight calculation was not considered in this study and therefore the extrapolation of conclusions would be limited to some extent.

### Conclusions

This paper indicates that occupational burnout among Chinese physicians is common in our studied samples. Role stress and social support are related to occupational burnout, but not all dimensions of both role stress and social support prove to be relevant factors affecting physicians’ occupational burnout. There may be partial mediation effects of social support, mainly through coworker support and family support, within the impact of role stress on occupational burnout among Chinese physicians. These findings signify that health sector managers should pay attention to physicians’ occupational burnout and the risks resulting from it. Efforts should be made to develop strategies to reduce role stress and to strengthen support from coworkers and family, thereby mitigating the risk of occupational burnout.
